# Serum Oxidant and Antioxidant Status Following an All-Out 21-km Run in Adolescent Runners Undergoing Professional Training—A One-Year Prospective Trial

**DOI:** 10.3390/ijms140715167

**Published:** 2013-07-22

**Authors:** Tom K. Tong, Zhaowei Kong, Hua Lin, Giuseppe Lippi, Haifeng Zhang, Jinlei Nie

**Affiliations:** 1 Dr. Stephen Hui Research Centre for Physical Recreation and Wellness, Department of Physical Education, Hong Kong Baptist University, Hong Kong, China; E-Mail: tongkk@hkbu.edu.hk; 2 Faculty of Education, University of Macau, Macao, China; E-Mail: zwkong@umac.mo; 3 College of Physical Education, Liaoning Normal University, Dalian 116029, Liaoning, China; E-Mail: wam3627459@163.com; 4 Laboratory of Clinical Chemistry and Hematology, Academic Hospital of Parma, Parma 43126, Italy; E-Mail: giuseppe.lippi@univr.it; 5 College of Physical Education, Hebei Normal University, Shijiazhuang 050024, Hebei, China; E-Mail: hbnuzhanghaifeng@sina.com; 6 School of Physical Education and Sports, Macao Polytechnic Institute, Macao, China

**Keywords:** adolescent, oxidative stress, antioxidant, endurance exercise

## Abstract

This study investigated the 1-year longitudinal effect of professional training in adolescent runners on redox balance during intense endurance exercise. Changes in selected serum oxidant and antioxidant status in response to a 21-km running time trial in 10 runners (15.5 ± 1.3 years) undergoing professional training were evaluated twice in 12 months (pre- and post-evaluation). Venous blood samples were collected immediately before and 4-h following the 21-km run for analysis of serum concentrations of thiobarbituric acid-reactive substances (TBARS), xanthine oxidase (XO), catalase (CAT), reduced glutathione (GSH), superoxide dismutase (SOD), and total antioxidant capacity (T-AOC). In pre-evaluation trial, serum TBARS and SOD decreased after the 21-km run (*p <* 0.05) while XO, GSH, CAT and TAOC were unchanged. In post-evaluation trial, serum TBARS and SOD decreased, whereas XO and CAT increased post-exercise (*p <* 0.05). Furthermore, pre-exercise serum T-AOC, post-exercise serum XO, CAT, T-AOC (*p <* 0.05), and GSH (*p* = 0.057) appeared to be higher than the corresponding pre-evaluation values. The current findings suggest that a professional training regime in adolescent runners is not likely to jeopardize the development of their antioxidant defense. However, uncertainties in the maintenance of redox balance in runners facing increased exercise-induced oxidative stress as a consequence of training-induced enhancement of exercise capacity await further elucidation.

## 1. Introduction

It is well established that endurance exercise is accompanied by an increased V̇O_2_, which, in turn, may trigger reactive oxygen species (ROS) production [1]. Although ROS have a prominent role in cell damage, their signaling function is important in mediating various physiological responses or adaptations to exercise, including glycogen repletion, myokine production, and up-regulation of antioxidant defense mechanisms [2–4]. These positive effects on cells are the result of a balance between ROS production and antioxidant defense. When the ROS exceed available antioxidants, however, oxidative stress may emerge. Long-term exposure to oxidative stress results in protein and lipid oxidation, DNA damage, and ultimately apoptosis. These unfavorable changes are associated with cell aging, and participate in certain degenerative pathologies, such as cardiovascular and metabolic disorders [1,5]. On the other hand, a protracted increase in ROS concentration in muscle cells following high-volume intense endurance training in athletes with inadequate recovery may lead to alterations of mitochondrial function and associated respiratory complexes, contractile dysfunction, as well as membrane potential perturbation [6–8]. As a result, reduction of muscular force-generating capacity, post-exercise muscular damage and associated pain may occur. The repetitive muscular ROS-induced loss of contractile function is supposed to predispose to overtraining syndrome, and eventually impair whole-body endurance performance [1].

Year-round training in professional endurance sports, such as long-distance running and road cycling beginning at a relatively young age is increasingly frequent in youth, with a training volume that is globally comparable to that of adult athletes (*i.e*., 1.5 to 3 training h per session, two sessions per day, 6 days per week). One of arguments against this form of training is that adolescent athletes are more susceptible than adults to oxidative stress due to high-intensity endurance exercise, because their endogenous anti-oxidative defense system appears to be less efficient compared with adults [9]. Moreover, adolescents rely more on aerobic metabolism than adults when subjected to identical bulks of physical exercise [10,11]. Although well-maintained resting blood redox balance has been described in adolescent athletes participating in professional endurance training [12], impaired oxidant-antioxidant balance has been observed after a single session of exhaustive endurance training in athletes [13]. Previous findings pointed out that the antioxidant capacity of individuals might have a genetic basis [14]. Moreover, developmental changes in the immature endogenous anti-oxidative system are age-dependent [15], and may be modified by environmental factors such as nutritional deficiencies [16]. It is still unknown, however, whether adolescent athletes undergoing endurance training in a “professional” form would impair the development and function of their antioxidant defense system. Therefore, the aim of this study was to investigate the longitudinal effect of professional training in adolescent runners on their antioxidant defense against oxidative stress elicited during intense endurance exercise, by comparing two identical evaluations of serum redox balance in response to a 21-km running time trial in adolescent runners undergoing 1-year professional training.

## 2. Results and Discussion

### 2.1. Results

A significant improvement (*p* < 0.05) in the 21-km time-trial performance was observed in all runners. The group’s mean running time improved from 95.4 ± 10 min (range: 83–114 min) pre-evaluation to 85.2 ± 4.2 min (range: 77.3–90.3 min) post-evaluation, equivalent to 10.2% ± 5.5% of pre-evaluation values.

Table 1 shows changes in levels of serum thiobarbituric acid-reactive substances (TBARS), xanthine oxidase (XO), reduced glutathione (GSH), catalase (CAT), total antioxidant capacity (T-AOC), and superoxide dismutase (SOD) induced by the maximum 21-km run during the pre- and post-evaluation trials.

For pre-exercise oxidant and anti-oxidant levels, only serum T-AOC (*p* < 0.05) was significantly increased post-evaluation. No statistically significant variation was observed in other variables (*p* > 0.05). After completing the 21-km run in the pre-evaluation trial, serum TBARS and SOD were reduced from pre-exercise levels (*p* < 0.05), whereas the remaining parameters were unchanged (*p* > 0.05). In the post-evaluation trial, post-exercise serum TBARS and SOD were also reduced, while serum XO and CAT were significantly increased from the corresponding pre-exercise values (*p* < 0.05). Moreover, post-exercise levels of serum XO, CAT, and T-AOC were significantly (*p* < 0.05) higher, and serum GSH also tended to be higher (*p* = 0.057), than the corresponding pre-evaluation values.

**Figure 1:**
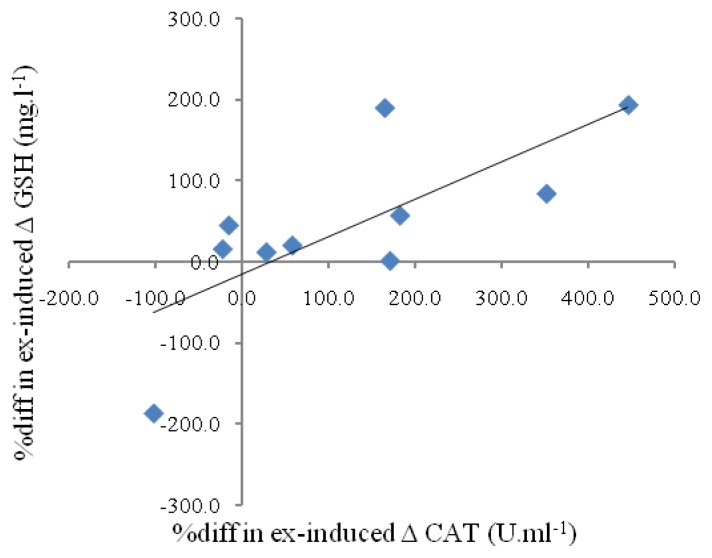
The linear relationship *(r =* 0.74, *n =* 10, *p* < 0.05) between differences in the exercise-induced alteration (Δ) in serum GSH and CAT resulting from the yearly training program expressed as a percentage of pre-evaluation values.

As regards the inter-individual relationship among the changes in pre-exercise serum redox status induced by the yearly training program, significant correlations (*p* < 0.05) were observed between GSH and CAT (*r* = 0.82); GSH and SOD (*r* = 0.65); and CAT and SOD (*r* = 0.70), when the changes in the serum variables were expressed as a percentage of pre-evaluation values. For differences in exercise-induced change in serum GSH, CAT and XO resulting from the yearly training program expressed as a percentage of pre-evaluation values, significant correlations (*p* < 0.05) were also found between GSH and CAT (*r* = 0.74) (see Figure 1), GSH and OX (*r* = 0.78), and CAT and OX (*r* = 0.67).

### 2.2. Discussion

The present study investigated the 1-year longitudinal effect of professional training in adolescent runners on acute changes in serum redox status in response to a 21-km running time trial. To the best of our knowledge, this is the first study to assess whether or not the endurance training of adolescent athletes performed according to a professional profile interferes with development of their antioxidant capacity for counteracting the burst of ROS generated during intense endurance exercise. The present study is a follow-up of our previous investigation of resting blood redox balance of professional adolescent athletes [12]. According to our current and previous findings, adolescent athletes participating in professional endurance sports training, with a training volume comparable to that of adult athletes, do not show evidence of inferior development in their antioxidant defense system. Although this study involved both male and female runners, we did not aim at a gender comparison of exercise-induced oxidative stress due to the limited sample size. In fact, gender effects on exercise-induced alterations in blood redox biomarkers were proven to be insignificant when exercise was performed with comparable intensity and duration [17].

Prior to this investigation, the adolescent runners had participated in professional training for an average of ~1.5 years. By comparison with data of untrained adolescents of a similar age in our recent study [12], higher pre-exercise serum antioxidant status was observed in adolescent runners, and this fact is at least in part attributable to the dose-dependent mechanism of adaptation to chronic exercise-induced increase in ROS formation [5]. Compared with the pre-evaluation level, serum TBARS was reduced after the 21-km trial run in a racing mode, but it was not accompanied by any increase in other serum variables at the time point of 4 h after exercise. Although the TBARS assay detects the secondary product of lipid peroxidation, the decrease in serum TBARS post-exercise may reflect to a certain extent the lack of oxidative stress in runners during the endurance run [3]. However, the lack of increase in serum CAT was in contrast to the modifications in the enzymatic antioxidant activities previously reported in humans and animals after aerobic exercise [1]. Beside the decrease in post-exercise serum SOD, the current serum antioxidant profile may reveal somewhat the inadequacy of endogenous antioxidant enzyme production in adolescent athletes, in the face of the challenge of ROS generation during prolonged exercise [18]. The absence of oxidative stress in this case could plausibly have resulted from the sufficient availability of non-enzymatic antioxidants, which were not specifically assessed in the present study. Nevertheless, we observed a trend of decrease in serum GSH post-exercise in the pre-evaluation trial, which was in agreement with previous findings of GSH reduction during aerobic exercise due to its consumption for counterbalancing ROS generation [19]. Another possibility for this lack of increase in antioxidant activities after the 21-km run may involve the specific redox response to anaerobic exercise [1]. One may observe that the 21-km run was carried out in a racing mode in the present study, with runners running at high speed and in an anaerobic condition in the last one or two laps before the end of the trial. In fact, the current findings of responses in serum antioxidant status to the time trial were in agreement with the unchanged or reduced antioxidant activities resulting from anaerobic-type exercise or intermittent-type sport with mixed aerobic and anaerobic exercise bouts reported in previous studies [1,20].

With runners going through 12-month professional training, the levels of pre-exercise serum TBARS and XO were maintained. No marked degradation in pre-exercise serum antioxidant status was observed, whereas a slightly but significant increase was observed in serum T-AOC post-evaluation. It is known that antioxidant status and oxidative stress evolve during a sport season, being mostly periodized according to training load and competitions [21]. The current post-evaluation findings in pre-exercise redox status, and so as the post-exercise data, are the final outcomes of the yearly training program in which the adolescent runners were involved. Nonetheless, the well-maintained pre-exercise redox balance does support our previous notion that the participation of adolescent athletes in professional endurance sports training, with a training volume comparable to that of adult athletes, is not likely to be associated with chronic impairment in resting blood redox balance [12]. Furthermore, the increase in pre-evaluation antioxidant status was only found in serum T-AOC, and not in other biomarkers. This is in agreement with previous evidence showing that the consequent adaptive responses of the antioxidant system to exercise training are usually pronounced in subjects with low training level at beginning of the protocol [1]. In fact, T-AOC can be considered a biomarker that reliably reflects the combined capacity of antioxidants in serum, so that its increase in serum may simply result from nutritional effects rather than from adaptation of oxidative stress [22].

A decrease in serum TBARS and an increase in serum CAT levels were also observed after repeating the 21-km run time trial post-evaluation. The decreased serum TBARS revealed the lack of exercise-induced oxidative stress, which might be partly attributable to increased production of antioxidant enzyme(s) of CAT. In line with our previous findings [12], the changes in exercise-induced alterations in serum GSH and CAT resulting from the yearly training program in the present study are correlated (Figure 1). The current findings suggest that the lack of oxidative stress post-exercise in adolescent runners might result from the integrative effect of enhancement of individual antioxidants as an adaptive mechanism to professional endurance training. It is noteworthy that the marked correlations observed (*r* ≥ 0.65) among alterations in pre-exercise serum GSH, CAT and SOD resulting from the yearly training further support this hypothesis, thus corroborating the notion that antioxidant defenses in human may interplay as a coordinated system, with various metabolites and enzymes having synergistic and interdependent effects. Hence, the defensive strength of an individual antioxidant against ROS damage may depend on the proper function of other members of the system [23,24].

On the other hand, we observed that serum XO increased significantly in runners in response to the 21-km run in the post-evaluation trial. An increase in XO activity usually takes place in two scenarios, including enhanced catabolism of purins and tissue ischaemic reperfusion/reoxygenation, which appear in active muscles of athletes experiencing tremendous physical exertion and anaerobic energy demand [25]. In the post-evaluation 21-km time trial, the performance of runners had improved by approximately 10% in comparison with the pre-evaluation time trial. It is reasonable to put forward the hypothesis that the increase in post-exercise serum XO might have been at least partially due to the enhanced anaerobic contribution to the performance of a high-speed run when runners approached the end of the trial. Moreover, the concomitant decrease in post-exercise serum SOD is in agreement with previous findings of a decline in enzymatic activity after a 30-s all-out exercise [26]. The decline in SOD after all-out exercise, which has been partly attributed to the inhibition of enzymatic activity in the presence of H_2_O_2_[27], might be a result of SOD defense against XO catalyzed-superoxide radical production. In the present study, despite the post-exercise serum XO activity being increased in runners, the change in exercise-induced alteration in serum XO resulting from the yearly training was correlated with that in serum CAT and GSH (*r* ≥ 0.67). This suggests that the potential increase in XO-catalyzed ROS generation following the run might have been counterbalanced by corresponding increases in serum CAT and GSH. In fact, a similar plasma redox profile has recently been reported in professional adult handball players undergoing 1 year of competition and training. The lack of hyperoxidative state that normally follows high-intensity exercise in athletes was considerably associated with their adaptation to exercise-induced ROS production, as well as specifically designed training periodization [28]. Nevertheless, the implications of the current findings of an increase in post-exercise serum XO activity (associated with potential insufficiency of the antioxidant defense system) should not be neglected. Further investigations with additional biomarkers may provide explicit information about the ability of the antioxidant system of professional adolescent runners to counteract the increase in ROS with exercise.

Despite the fact that our findings provide reasonable information regarding development trends in defense against exercise-induced oxidative stress of professional adolescent runners, further interpretation is limited by, firstly, the challenge of comparison with untrained adolescents who were capable of completing a 21-km run. Also, changes in selected blood redox markers after the time trial were assumed to reflect the true alterations of the oxidant and antioxidant status in active tissues. It is due to the fact that tissue biopsy sampling techniques in humans can be only performed in special cases. Nevertheless, several redox biomarkers in blood (e.g., TBARS, CAT, and GSH) have been proven as a reliable index that mirrors the exercise-induced changes that appear in the same markers in skeletal muscle, heart, and liver [29]. It is also noteworthy that the changes in the limited blood redox markers may not fully reflect the true alterations of the oxidant and antioxidant status after the time trial, since runners ran at mixed aerobic and anaerobic intensities. Moreover, the TBARS assay is not specific to the lipid peroxidation product. There are some reservations about the validity of the assay in detecting lipid peroxidation. Despite this, exercise-induced increase in TBARS has been repeatedly reported in previous studies [13,30,31], and the changes in TBARS concentrations were reported to be similar to changes in F2-isoprostane concentrations after exercise [32]. In future studies, the assessment of additional biomarkers, such as plasma F2-isoprostanes, plasma antioxidant vitamins, Trolox-equivalent antioxidant capacity, as well as uric acid may provide additional information about exercise-induced oxidative stress in athletes. Finally, the changes observed in female runners were independent of their menstrual cycle. The expectable differences in estrogen level and resultant alterations in antioxidant status in the runners might have contributed to the current findings of exercise-induced changes in redox status. The control of menstrual cycle influences in female athletes in future studies might be advisable, although the influence of changes in estrogen level during the menstrual cycle on exercise-induced oxidative stress is considered of minor significance in young women [33].

## 3. Experimental Section

### 3.1. Subjects

The study population consisted of seven female and three male adolescent long-distance runners (age range: 13.9–17.3 years, mean age ± SD: 15.5 ± 1.3 years, Tanner stage: 2–4, 3.3 ± 0.8, height: 170.4 ± 8.0 cm, weight: 54.4 ± 8.8 kg), who had trained for a mean period of 1.4 ± 1.0 years at a local running club and performed at national level. All runners had no familial history of cardiovascular disease or assumed related medication. Moreover, none of them had received anti-inflammatory medications or any form of nutritional supplements. Following an explanation of the purpose and constraints of the investigation, the adolescent runners and their tutors gave written informed consent for participating in this study. The local Ethical Committee for the Use of Human & Animal Subjects in Research provided ethical approval of the study.

### 3.2. Professional Long-Distance Run Training

The yearly training programs of the runners were periodized according to different segments of the training year. The investigations started at the beginning of the preseason segment, and were completed at the end of off-season. Coaches and runners were blinded to the investigation. The training programs adopted by runners during the investigation period were profession-oriented, and were not manipulated for the purpose of this investigation. In general, the training programs of the runners consisted of 2 × 1.5- to 2-hour sessions per day, 6.5 days per week. The distance covered during training was 7–21 km per day, 60–80 km per week. The training regimes were gender-specific, but identical within each gender. All the training activities were held at the training camp of a local sports club, in Liaoning province, China. The room and board of runners were uniform and provided by the sports club. The diets of the athletes were well controlled and under close surveillance at the camp. During in-season, the runners participated in races mostly held locally or in neighboring provinces.

### 3.3. Study Design

We evaluated the alterations in serum redox status in response to an all-out 21-km run in adolescent runners undergoing 1-year professional training. The oxidant and antioxidant status was determined by quantifying serum concentrations of TBARS, GSH, and T-AOC, as well as the enzymatic activity of XO, SOD, and CAT. Runners’ diets throughout pre- and post-evaluation blood sampling were similar.

### 3.4. Procedures

Experimental trials: prior to the yearly training, all runners were requested to perform a 21-km running time-trial with maximum effort. To minimize the negative effect of boredom during the running task, all subjects completed the running test at the same time, in one trial in a racing mode, on a standard 400-meter track. Upon arrival at the testing station, a 10-min rest period was observed. Following the rest period, pre-exercise venous blood samples for biochemical analyses were immediately collected prior to warm-up exercise. During the 21-km time-trial, the running speed was self-selected. Water replacement ad libitum was voluntarily taken at 5-km intervals. The distance in km covered during the test was displayed to subjects on completion of each lap. After the time-trial, blood samples were taken at a time point of 4 h post-exercise. For this group of adolescent runners who engaged in routine training of long-distance runs two times a day, the periods of 4 h post-exercise were considered as the early recovery period, thus enabling a relatively stable point for observing oxidative stress [13]. Upon completion of the yearly training, the 21-km time-trial and associated pre- and post-exercise blood samplings were repeated.

Prior to the time-trials, the runners were requested to maintain their usual dietary and hydration habits, and refrain from intense physical training for not less than 24 h. During the time-trials, the ambient temperature was around 5 °C, and the relative humidity was approximately 35%.

### 3.5. Serum Measurements

After blood sampling in evacuated blood tubes containing no additives, serum was separated at 2000 g for 20 min, aliquoted and stored at −20 °C for later analysis.

The levels of TBARS and GSH and enzymatic activity of XO, SOD, and CAT were measured using commercial assay kits (Nanjing Jiancheng Institute, Nanjing, China) on a spectrophotometer (DU7400, Beckman Co, Fullerton, CA, USA), according to manufacturers’ instructions. Briefly, lipid peroxidation was evaluated using the TBARS method and was expressed as a TBARS concentration. This method was used to obtain a spectrophotometric measurement of the color produced during the reaction of thiobarbituric acid and malondialdehyde (an indicator of peroxidation of polyunsaturated fatty acids in cell membranes subsequent to reactions with ROS) at 535 nm. The TBARS level was expressed as nmol.mL^−1^. The GSH level was assessed with a colorimetric assay at 412 nm following reaction with DTNB [5,5′-Dithio-Bis (2-Nitrobenzoic Acid)], and was expressed as nmol·mL^−1^. The activities of XO and SOD were measured by the xanthine-xanthine oxidase system, which is a superoxide anion generator, following the increase or decrease of absorbance, respectively. The activity of XO and SOD was expressed as U·L^−1^ and U·mL^−1^. CAT activity, expressed as U·mL^−1^, was determined by the decrease in H_2_O_2_ absorbance at 240 nm. T-AOC was measured by the ferric reducing ability of plasma (FRAP) assay of Benzie and Strain [34]. The stable color of the Fe^2+^-*O*-phenanthroline complex (produced with reducing agents in plasma by reducing Fe^3+^ to Fe^2+^, which reacts with the substrate *O*-phenanthroline) was measured at 520 nm. T-AOC was expressed in U·mL^−1^, where 1 unit is defined as an increase in absorbance (A520) of 0.01 per min at 37 °C.

The inter- and intra-assay coefficients of variation of the biochemical analyses were as follows: TBARS, 5.4% and 2.2%; GSH, 4.8% and 1.8%; XO, 9.2% and 4.5%; SOD, 8.7% and 5.0%; CAT, 11.4% and 6.2%; and T-AOC, 8.5% and 4.6%, respectively.

### 3.6. Statistical Analysis

Kolmogorov-Smirnov normality test revealed that the data for all the variables was normally distributed. Two-way ANOVA with repeated measures was computed to assess differences in TBARS, GSH, XO, SOD, CAT and T-AOC between time points (pre- *vs.* post-exercise), and across yearly training (pre- *vs.* post-evaluation). Post-hoc analyses using the Newman-Keuls were performed for cases in which the main effect was significant. The relationships between variables were assessed using simple regression. All tests for statistical significance were standardized at an alpha level of *p <* 0.05, and all results were expressed as mean ± SD.

## 4. Conclusions

In summary, reductions in serum activities of TBARS and SOD in response to a pre-evaluation 21-km running time trial were found in professional adolescent runners. After the yearly training, similar results in post-exercise serum TBARS and SOD, along with significant increases in post-exercise serum CAT and XO, were observed. Moreover, the alterations in pre-exercise activities of serum GSH, CAT and SOD, as well as changes in the exercise-induced alterations in the serum GSH, CAT and XO, resulting from the yearly training were significantly associated. In light of our current and previous findings [12], a professional training regime in adolescent runners, with a training volume comparable to that of adult athletes, is not likely to jeopardize the development of their antioxidant defense system for counteracting ROS generation during endurance exercise. Conversely, enhanced antioxidant capacity would be gained in adaptation to long-term training. Nevertheless, further investigations with a larger sample size and additional biomarkers may be needed for clarifying the existing uncertainties in the maintenance of redox balance in professional adolescent runners when facing the increased challenge of exercise-induced oxidative stress as a consequence of training-induced enhancement of exercise capacity.

## Figures and Tables

**Table 1:** Changes in the levels of serum thiobarbituric acid-reactive substances (TBARS), xanthine oxidase (XO), reduced glutathione (GSH), catalase (CAT), total antioxidant capacity (T-AOC), and superoxide dismutase (SOD) induced by the maximum 21-km run during the pre- and post-evaluation trials (*n =* 10).

		Pre-evaluation	Post-evaluation
TBARS (nmol·mL^−1^)	Pre-ex	5.64 ± 0.92	5.61 ± 0.91
	Post-ex	4.24 ± 0.85 *	5.40 ± 1.27 *

XO (U·L^−1^)	Pre-ex	14.7 ± 2.9	16.4 ± 4.5
	Post-ex	13.4 ± 3.9	24.4 ± 7.8 *,^a^

GSH (mg·L^−1^)	Pre-ex	20.9 ± 10.3	21.1 ± 8.9
	Post-ex	10.1 ± 4.7	21.1 ± 12.7

CAT (U·mL^−1^)	Pre-ex	4.48 ± 1.51	3.84 ± 1.63
	Post-ex	3.12 ± 4.19	7.79 ± 2.79 *,^a^

T-AOC (U·mL^−1^)	Pre-ex	11.2 ± 1.92	13.3 ± 1.0 a
	Post-ex	12.3 ± 1.33	13.7 ± 1.59 a

SOD (U·mL^−1^)	Pre-ex	69.2 ± 12.3	67.0 ± 13.5
	Post-ex	63.4 ± 15.6 *	60.9 ± 13.9 *

Data are mean ± SD;

* Significantly different from corresponding pre-ex value, *p* < 0.05;

a Significantly different from corresponding pre-evaluation value, *p* < 0.05;

TBARS, thiobarbituric acid-reactive substances; XO, xanthine oxidase; GSH, reduced glutathione; CAT, catalase; T-AOC, total antioxidant capacity; SOD, superoxide dismutase; pre-ex, pre-exercise; post-ex, post-exercise.
